# Psilocybin for treatment-resistant depression without psychedelic effects: study protocol for a 4-week, double-blind, proof-of-concept randomised controlled trial

**DOI:** 10.1192/bjo.2023.535

**Published:** 2023-07-25

**Authors:** Muhammad Ishrat Husain, Daniel M. Blumberger, David J. Castle, Nicole Ledwos, Elise Fellows, Brett D. M. Jones, Abigail Ortiz, Stefan Kloiber, Wei Wang, Joshua D. Rosenblat, Benoit H. Mulsant

**Affiliations:** Campbell Family Mental Health Research Institute, Centre for Addiction and Mental Health, Canada; and Department of Psychiatry, Temerty Faculty of Medicine, University of Toronto, Canada; Department of Psychiatry, Temerty Faculty of Medicine, University of Toronto, Canada; and Temerty Centre for Therapeutic Brain Intervention, Centre for Addiction and Mental Health, Canada; Department of Psychiatry, University of Tasmania, Australia; General Adult Psychiatry and Health Systems Division, Centre for Addiction and Mental Health, Canada; General Adult Psychiatry and Health Systems Division, Centre for Addiction and Mental Health, Canada; and Institute of Medical Science, Temerty Faculty of Medicine, University of Toronto, Canada; Centre for Complex Interventions, Centre for Addiction and Mental Health, Canada; and College of Public Health, University of South Florida, USA; Mood Disorder Psychopharmacology Program, Poul Hansen Depression Centre, Unit University Health Network, Canada

**Keywords:** Treatment-resistant depression, major depressive disorder, psilocybin, randomised controlled trial, psychedelic-assisted psychotherapy

## Abstract

**Background:**

Randomised controlled trials (RCTs) of psilocybin have reported large antidepressant effects in adults with major depressive disorder and treatment-resistant depression (TRD). Given psilocybin's psychedelic effects, all published studies have included psychological support. These effects depend on serotonin 2A (5-HT2A) receptor activation, which can be blocked by 5-HT2A receptor antagonists like ketanserin or risperidone. In an animal model of depression, ketanserin followed by psilocybin had similar symptomatic effects as psilocybin alone.

**Aims:**

To conduct a proof-of-concept RCT to (a) establish feasibility and tolerability of combining psilocybin and risperidone in adults with TRD, (b) show that this combination blocks the psychedelic effects of psilocybin and (c) provide pilot data on the antidepressant effect of this combination (compared with psilocybin alone).

**Method:**

In a 4-week, three-arm, ‘double dummy’ trial, 60 adults with TRD will be randomised to psilocybin 25 mg plus risperidone 1 mg, psilocybin 25 mg plus placebo, or placebo plus risperidone 1 mg. All participants will receive 12 h of manualised psychotherapy. Measures of feasibility will include recruitment and retention rates; tolerability and safety will be assessed by rates of drop-out attributed to adverse events and rates of serious adverse events. The 5-Dimensional Altered States of Consciousness Rating Scale will be a secondary outcome measure.

**Results:**

This trial will advance the understanding of psilocybin's mechanism of antidepressant action.

**Conclusions:**

This line of research could increase acceptability and access to psilocybin as a novel treatment for TRD without the need for a psychedelic experience and continuous monitoring.

Treatment-resistant depression (TRD) is typically defined as a major depressive episode that does not respond to two or more adequate antidepressant trials, or a relapse or recurrence of a major depressive episode during treatment.^1,2^ TRD affects up to a third of all individuals with major depressive disorder (MDD).^3^ TRD is associated with a significant decline in social and occupational functioning and higher rates of death by suicide and all-cause mortality than MDD that responds to treatment.^4^ Persistent symptoms in TRD often translate into substantial work loss, healthcare resource utilisation and costs.^5^ Current pharmacotherapy for TRD, including augmentation of antidepressants with atypical antipsychotics, lithium or ketamine, have high rates of non-response^6^ and can be associated with problematic adverse effects (e.g. sedation, weight gain, diabetes, tardive dyskinesia), leading to non-adherence.^7^ Electroconvulsive therapy (ECT) is the most efficacious intervention for TRD. However, only a minority of patients with TRD receive ECT because of the associated stigma, lack of access and fears of cognitive adverse effects.^8^ Repetitive transcranial magnetic stimulation is a potential alternative to ECT, but its response and remission rates are similar to pharmacotherapy,^6^ leaving a large proportion of patients with TRD in need of novel interventions.

## Psilocybin for the treatment of depression

During the past decade, there has been a resurgence of interest in psychedelic compounds as novel treatments for mental disorders, including TRD.^9^ In particular, psilocybin (the chemical component of ‘magic mushrooms’), in conjunction with supportive psychotherapy, has shown large and sustained antidepressant effects in patients with MDD and TRD in some contemporary open-label and randomised controlled trials (RCTs).^10–12^ For instance, in an open-label trial of psilocybin-assisted psychotherapy (PAP), 63% of 19 participants with TRD responded 1 week after treatment, and 32% did not require any antidepressant or therapy for a further year.^11^ Similarly, an RCT of 24 participants with MDD showed large effect sizes for PAP after 1 week (Cohen's *d* = 2.5) and 4 weeks (Cohen's *d* = 2.6) post-treatment, compared with the waitlist control.^12^ A long-term follow-up study of the same participants showed response and remission rates of 75% and 58%, respectively, at 12 months.^13^ In addition, a trial comparing PAP with escitalopram in 59 participants with non-resistant MDD showed that PAP with two doses of psilocybin was as effective as 6 weeks of escitalopram in reducing depressive symptoms.^10^ Most recently, a phase 2 RCT of PAP in 233 patients with TRD indicated that 25 mg psilocybin led to a significant reduction in scores on the Montgomery–Asberg Depression Rating Scale (MADRS) for at least 6 weeks post-treatment, compared with 1 mg psilocybin (an active placebo), without significant differences in serious adverse events between groups.^14^

## Does psilocybin require psychedelic effects to treat depression?

However, most published trials to date are limited by small sample sizes, inadequate control groups, lack of blinding or highly selected participants.^15,16^ Despite these methodological limitations, the results of these trials have led to growing enthusiasm for the use of psilocybin for the treatment of MDD and related disorders. Currently, it is assumed that psilocybin's therapeutic effects in MDD require altered consciousness (i.e. the psychedelic ‘trip’), which is dependent on serotonin 2A (5-HT2A) receptor activation.^17^ However, this assumption is based on evidence from a series of trials in which all participants treated with the standard efficacious dosage of psilocybin experienced its psychedelic effects, which prevented real blinding.^18^ Psilocybin is a tryptamine, chemically similar to the amino acid tryptophan, and the neurotransmitter serotonin.^19^ It is a prodrug for psilocin, which readily crosses the blood–brain barrier and acts as a potent partial agonist at serotonin 5-HT2A and 2C receptors.^19,20^ As a 5-HT2A/2C agonist, psilocin is regarded as a ‘classic’ psychedelic, which elicits altered states of consciousness, consisting of visual and sometimes auditory effects, changes in perception, distortions of time and a range of ‘mystical experiences’, including a sense of awe, unity and empathy.^19^ These mystical experiences have been correlated with improvement in mood in healthy volunteers and palliative care patients with end-of-life distress.^19^ However, there is no evidence that these psychedelic effects are the cause of the antidepressant effect in patients with MDD.^19^ In fact, in a recent study, 24 participants who received PAP for MDD showed no significant associations between subjective psychedelic effects and sustained improvement in depressive symptoms, suggesting that psychedelic effects may not be necessary to harness psilocybin's antidepressant effects.^13^

In its current form, PAP is not scalable for widescale clinical adoption. All contemporary clinical trials have investigated psilocybin in the context of several hours of psychological support, given its highly potent psychedelic effects and the need for continuous monitoring. The psychotherapy involves at least 2 h of preparatory sessions, 8 h of supportive therapy during the dosing session in the presence of two trained therapists, and 2 h of post-dosing integration sessions. This amount of specialised psychotherapy impedes scalability of psilocybin as an intervention for MDD and TRD, given the limited resources and access to therapists to deliver PAP in most jurisdictions. In addition, determining whether psilocybin's psychedelic effects are necessary for its antidepressant effects could increase its acceptability in patients apprehensive about the psychedelic ‘trip’ and its associated effects. In humans, the intensity of psilocybin-induced perceptual changes correlates with 5-HT2A activation.^21^ However, psilocybin's antidepressant effects may be mediated through rapid activation of other critical 5-HT receptors. Two 5-HT2A receptor antagonists – ketanserin (an antihypertensive) and risperidone (an atypical antipsychotic) – have been shown to eliminate self-reported psilocybin psychedelic effects in humans.^22^ In a recent pre-clinical study of a mouse model of depression that used chronically stressed mice, psilocybin reversed anhedonic responses and this anti-anhedonic effect was not prevented by pre-treatment with ketanserin.^23^ To our knowledge, no study has investigated in humans whether psilocybin's antidepressant effects are dependent on its activation of the 5-HT2A receptor (and hence on its psychedelic effects). To address this clinically relevant question, we have designed a proof-of-concept RCT to establish the feasibility, tolerability and preliminary efficacy of combining psilocybin (25 mg) with risperidone (1 mg) in patients with TRD. This trial will assess the hypothesis that pre-treatment with risperidone blocks the psychedelic effects of psilocybin. In addition, it will explore the antidepressant effect of psilocybin plus risperidone, compared with psilocybin alone.

## Method

This is a single-site, phase 2, 4-week, double-blind, placebo-controlled randomised trial, with all procedures taking place at the Centre for Addiction and Mental Health (CAMH), an academic hospital in Toronto, Ontario, Canada. In a parallel-group design, 60 adults with a diagnosis of MDD as defined by the DSM-5,^24^ and a current major depressive episode that has not responded to at least two adequate trials of antidepressants, will be randomised with a 1:1:1 allocation ratio to one of three groups: 1 mg risperidone plus 25 mg of psilocybin, placebo risperidone plus 25 mg psilocybin, or 1 mg risperidone plus placebo psilocybin. A placebo plus placebo arm was not included for two reasons: to optimise allocation concealment, we favoured the use of an ‘active placebo’; also, we decided not to use a classic 2 × 2 design, to improve the efficiency of a study focused on patients with TRD. The expected mild psychoactive effects of risperidone (e.g. drowsiness, dysphoria) in those receiving risperidone plus either psilocybin or placebo will enhance blinding.

**Fig. 1:**
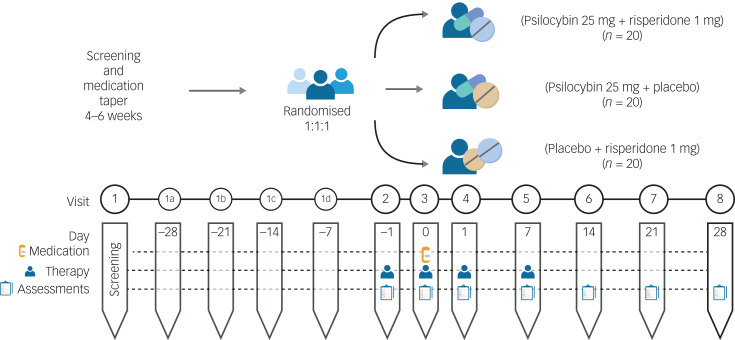
Study schematic.

**Fig. 2:**
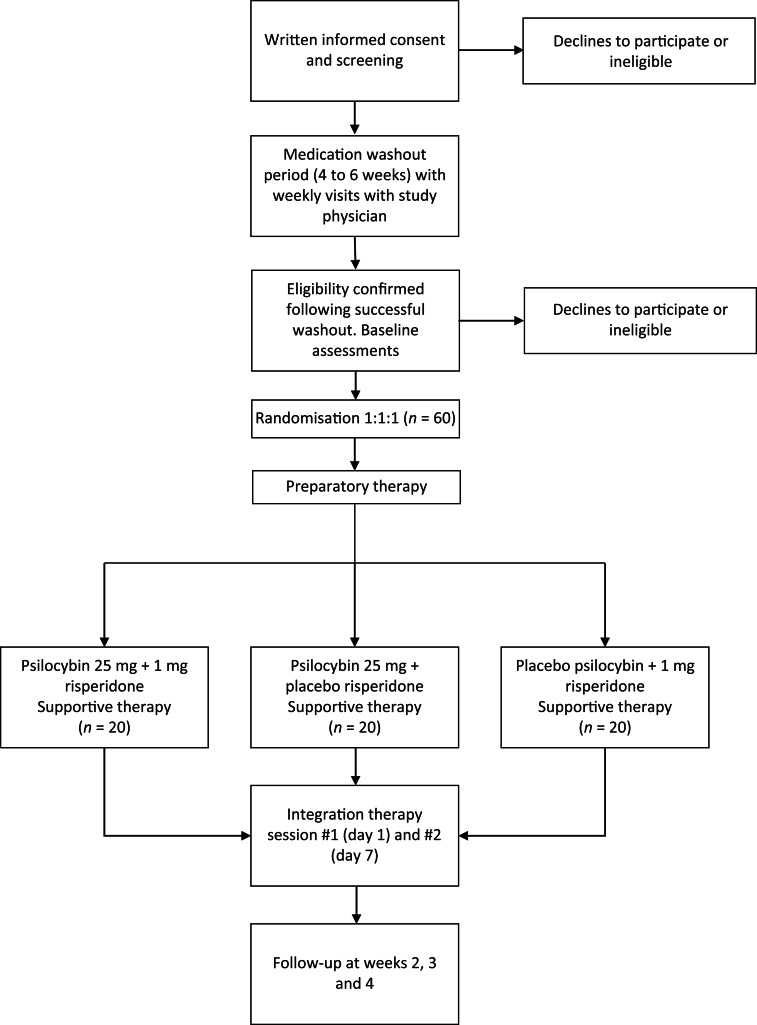
CONSORT flow diagram.

The 1 mg risperidone and matched placebo risperidone will be provided and packaged by CAMH pharmacy. The 25 mg psilocybin and matched placebo psilocybin will be provided by Filament Health (Burnaby, British Columbia, Canada). The psilocybin provided by Filament Health is manufactured according to good manufacturing practice guidelines. The capsules contain naturally extracted psilocybin. A study schematic is depicted in Fig. 1 and a planned Consolidated Standards of Reporting Trials (CONSORT) flow diagram is shown in Fig. 2.

We are using the atypical antipsychotic risperidone, a potent blocker of the 5-HT2A receptor, because a previous study in healthy volunteers has demonstrated that 1 mg risperidone effectively blocks psilocybin's psychedelic effects.^22^ In addition, risperidone is a readily available, safe and inexpensive generic drug that is widely used by psychiatrists for the treatment of TRD and other mental disorders. Hence, it could easily be combined with psilocybin in clinical practice if the combination was shown to be safe and effective. By contrast, other 5-HT2A antagonists, such as ketanserin or pimavanserin (an antipsychotic approved for the treatment of Parkinson's disease psychosis), are expensive and have limited data on their safety in MDD. Although risperidone also blocks dopaminergic receptors, psilocybin has no direct dopaminergic activity,^25^ and psilocybin's psychedelic effects are not blocked by first-generation D_2_ receptor antagonists like haloperidol.^22^

### Study procedures

#### Recruitment

Recruitment will occur via out-patient referrals from clinicians at CAMH and other hospitals in the Greater Toronto Area. All participants will be under the care of a study psychiatrist at CAMH to ensure clinical oversight during the trial; their usual treating psychiatrist will also be informed of their participation in the study. Potential participants who have expressed an interest in the study will be contacted by the study team for a pre-screening call, during which a description of the study will be given and a few questions regarding psychiatric and medical history will be asked. The pre-screening form will be reviewed by the Principal Investigator (M.I.H.), and participants who are preliminarily deemed eligible will be scheduled for a consent and screening visit.

#### Informed consent and screening

Written informed consent will be obtained by trained study personnel. Each participant will be provided with a copy of the informed consent form approved by the research ethics board (REB). Research personnel will explain the clinical trial to the potential participant and answer any questions that they may have; they can take as much time as they need to make their decision, and may consult with others (e.g. family members, other healthcare providers, etc.).

#### Eligibility criteria

The inclusion criteria are out-patient adults aged 18–65 years, with a primary diagnosis of non-psychotic MDD and a current major depressive episode as confirmed by the Structured Clinical Interview for DSM-5 (SCID-5),^26^ who meet the criteria for TRD. TRD is defined as a current major depressive episode (defined as a score of >14 on the 17-item Hamilton Rating Scale for Depression (HRSD))^27^ and no response to two or more antidepressant medications at an adequate dosage and duration (based on the Antidepressant Treatment History Form (ATHF)).^28^ Participants taking prescribed antidepressants or antipsychotics to manage symptoms will be required to taper off these medications over the screening period to be eligible for the study, as these medications can attenuate the acute effects of psilocybin. Participants must be able to demonstrate the capacity to provide informed consent, have the ability to read and communicate in English and be able to take oral medication. A complete list of inclusion and exclusion criteria is provided in Table 1.

**Table 1:** Inclusion and exclusion criteria

Inclusion criteria
Adult out-patients aged 18–65 years
Willingness to comply with all study procedures and agreement to lifestyle considerations throughout the study duration
Ability to read and communicate in English, such that their literacy and comprehension is sufficient for understanding the consent form and study questionnaires, as evaluated by study staff obtaining consent
Ability to take oral medication
All blood work within normal limits, including an eGFR > 40 mL/min/1.73 m^2^
Primary DSM-5 diagnosis of non-psychotic MDD, single or recurrent, based on the SCID-5 administered during the screening visit
Treatment-resistant depression defined as having a major depressive episode with a baseline HRSD score of >14 and no response to two or more trials of antidepressants at an adequate dosage and duration, based on the ATHF; there is no upper limit on the number of antidepressant treatment trials
Being off any antidepressant or antipsychotic medications for at least 2 weeks (or more depending on the medication) before baseline (visit 2) and for the duration of the study, with the participant's physician confirming that it is safe for the participant to do so
Being off any inhibitors of 5′-diphospho-glucuronosyltransferase (UGT)1A9 and 1A10, aldehyde dehydrogenase inhibitors and alcohol dehydrogenase inhibitors for at least 2 weeks (it can be longer depending on the medication) before baseline and for the duration of the study, with the participant's physician confirming that it is safe for the participant to do so
If the participant is capable of becoming pregnant, use of highly effective contraception for at least 1 month before screening and agreement to use such a method during study participation
Exclusion criteria
Pregnant as assessed by a urine pregnancy test at screening (visit 1) or baseline (visit 2), or intending to become pregnant during the study, or breastfeeding
Treatment with another investigational drugs or interventions within 30 days of screening or initiation of psychotherapy in the 12 weeks before screening
DSM-5 diagnosis of substance use disorder within the preceding 6 months (recreational use of tobacco, alcohol or cannabis, and prescribed opioids are permitted)
Active suicidal ideation with intent and plan as determined by item 3 of the HRSD
Any DSM-5 lifetime diagnosis (as determined by the SCID-5 clinical) of a schizophrenia spectrum disorder, other psychotic disorder (unless substance-induced or because of a medical condition), bipolar disorder type 1 or 2, obsessive–compulsive disorder or neurocognitive disorder Any DSM-5 lifetime diagnosis of paranoid or borderline personality disorder as determined by history
Any first-degree relative with a diagnosis of schizophrenia spectrum disorder, other psychotic disorder (unless substance-induced or because of a medical condition) or bipolar disorder type 1 or 2, as determined by interview of the participant and the family medical history form
Presence of a relative or absolute contraindication to psilocybin, including a drug allergy, recent stroke, uncontrolled hypertension, labile blood pressure, recent myocardial infarction, cardiac arrhythmia, severe coronary artery disease or moderate-to-severe renal or hepatic impairment
Presence of baseline prolonged QTc or *torsade de pointes* on the electrocardiogram, or history of long QTc syndrome or related risk factors
Lifetime use of serotonergic psychedelic drugs
Allergy or contraindication to risperidone, including insulin-dependent diabetes and history of hypoglycaemia on oral hypoglycaemic agent(s)
Any other clinically significant physical illness, including chronic infectious diseases, or any other concurrent illness that, in the opinion of the investigator, constitutes a health risk for the participant if they take part in the study or may interfere with the interpretation of the study results

eGFR, estimated glomerular filtration rate; MDD, major depressive disorder; SCID-5, Structured Clinical Interview for DSM-5; HRSD, Hamilton Rating Scale for Depression; ATHF, Antidepressant Treatment History Form; QTc, corrected QT interval.

#### Baseline (visit 2) and randomisation

Following the tapering of psychotropic medications, participants will attend a baseline visit (visit 2), during which clinical assessments and questionnaires will be administered. In addition, participants will engage in a 2-h preparatory therapy session with the study therapists (see below).

Each participant will be assigned a unique identification number once they have given informed consent and eligibility has been confirmed. The trial pharmacist will randomise participants to one of three groups in a 1:1:1 allocation: 1 mg risperidone plus 25 mg psilocybin, placebo plus 25 mg psilocybin, or 1 mg risperidone plus placebo. Randomisation will be computer-generated, using random permuted blocks with variable block sizes. The randomisation will be stratified based on severity of depressive symptoms (HRSD score ≤25 *v.* ≥26).^27^ After randomisation, the research pharmacist will dispense medication to a blinded study team member on the day of dosing, who will provide it to the participant. Allocation will be masked from the study investigators, co-investigators, study staff, therapists and participants, until all participants have completed the follow-up visits and the database has been cleaned and locked. To enhance blinding, efforts will be made to limit communication between therapists and study staff who rate the assessment measures. To assess the integrity of blinding procedures, participants and raters will be asked to complete a conventional guess form asking them whether they believe the participant received psilocybin alone, psilocybin with risperidone or risperidone alone, after they have completed all other research assessments at the week 2 visit. The research pharmacist will not be blinded; however, they will have no contact with the study team other than to provide the randomised investigational product to a study team member on the day of dosing. Emergency unblinding will be limited to situations when the Principal Investigator has determined that a participant's emergency care requires access to the treatment assignment. The study team will report any intentional or unintentional unblinding to the CAMH REB and Health Canada.

#### Dosing day (visit 3)

Dosing day (visit 3; day 0) will be scheduled following baseline (visit 2), after the participant has been deemed eligible to participate. Based on the treatment group to which the participant has been randomised, the participant will receive one of the following: 1 mg risperidone capsule followed 60 min later by 25 mg psilocybin capsule, placebo capsule followed 60 min later by 25 mg psilocybin capsule, or 1 mg risperidone capsule followed 60 min later by placebo capsule.

Each capsule will be taken orally with a glass of water under the supervision of a clinician; capsules will not be chewed or opened. After taking the second capsule, the participant will lie down on a bed. Therapists will encourage participants to focus their attention inward and stay with any experience that arises. To enhance inward focus, a preselected music playlist will be played quietly.

About 5–6 h after taking the second capsule, the participants will discuss their experience with the therapists. The participant will be discharged approximately 8 h after taking the second capsule, when, in the opinion of the study physician, the acute effects of the study drug have resolved.

#### Therapy

This study involves approximately 12 h of manualised therapy with trained study therapists. Therapists must be licensed by a regulatory body to provide therapy (e.g. College of Physicians and Surgeons of Ontario, College of Registered Psychotherapists of Ontario, Ontario College of Social Workers and Social Service Workers, etc.). Unlicensed therapists who are in the process of becoming licensed or those who have never administered PAP may participate in the trial as a therapist provided they are directly supervised by someone who is licensed. All therapists will undergo training with the Yale Manual of Psilocybin-Assisted Therapy of Depression^29^ as well as protocol-specific training for the study. Therapists will also receive ongoing peer supervision throughout the duration of the trial.

The participant will attend one preparatory session (visit 2) that occurs within 7 days before the dosing day (visit 3) to develop a therapeutic alliance, set intentions for the experience and learn what to expect on the dosing day. Ideally, the preparatory therapy session will occur the day before the dosing day. An additional preparatory session may be added at the discretion of the study investigator and therapists. The participant will also partake in two integration sessions 1 day (visit 4) and 1 week (visit 5) after the dosing day. PAP is closely related to acceptance and commitment therapy, with a focus on ‘psychological flexibility’, i.e. a person's ability to adapt to fluctuating situational demands; reconfigure mental resources; shift perspective and balance competing desires, needs and life domains. The therapist's role is to witness the participant's therapeutic process and provide unconditional positive regard during the experience. Therapy dyads are required, and efforts will be made to ensure that the same therapist dyad attends every therapy session.

#### Follow-up

Remote or in-person follow-up visits to assess depressive symptoms, anxiety, quality of life and well-being will occur 2, 3 and 4 weeks after the dosing day (visits 6, 7 and 8, respectively).

#### Outcome measures

The primary outcome measures will assess feasibility based on the recruitment rate in the trial and the overall completion rate for each of the three treatment groups. In addition, tolerability and safety will be assessed by comparing the rates of drop-outs attributed to adverse events and the rates of serious adverse events in the three groups.

The secondary outcome measure will be the total score on the 5-Dimensional Altered States of Consciousness Rating Scale (5D-ASC)^30^ at the end of the dosing day (visit 3), used to evaluate the psychedelic effects in each of the three groups. Participants will complete this self-report questionnaire at the end of the dosing day (visit 3), when the acute effects of the drug have worn off. The exploratory outcome measure will be the MADRS,^31^ used to evaluate antidepressant effects in the three groups at baseline and visits 4–8. The MADRS will be completed by raters who are blind to treatment allocation.

Other outcome measures will include the following: the Clinical Global Impressions scale,^32^ an overall measure of illness severity; the seven-item Generalised Anxiety Disorder-7,^33^ a self-report measure of severity for generalised anxiety symptoms; the Snaith–Hamilton Anhedonia Scale,^34^ a self-rated questionnaire assessing anhedonic symptoms; the World Health Organization Quality of Life Questionnaire,^35^ a self-rated measure of quality of life; and the Warwick–Edinburgh Mental Wellbeing Scale,^36^ a brief questionnaire to assess well-being. To assess expectancy bias, we will use the Stanford Expectations of Treatment Scale (SETS), a validated tool for measuring patient outcome expectancy in clinical trials.^37^ The SETS will be administered at the baseline visit.

### Statistical analysis

#### Sample size and power calculation

The proposed sample size (*N* = 60, with *n* = 20 in each of the three treatment groups) is based on published recommendations for pilot trials.^38^ This sample size will provide informative confidence interval estimates for continuous and binary outcomes. For example, the margins of error are 8.9% for recruitment rate and 7.6% for retention rate. For continuous outcomes, the margin of error is approximately 0.66 s.d. for between-group differences. For the secondary aim, we will have sufficient power (0.80) to detect an effect size of 0.96 (Cohen's *d*) for the psychedelic effects measured by the 5D-ASC, when comparing the psilocybin plus placebo group with the psilocybin plus risperidone or placebo plus risperidone groups. Previous RCTs in healthy volunteers have reported larger effects on the 5D-ASC when comparing psilocybin alone with psilocybin plus a 5HTA receptor antagonist.^22^

For the exploratory aim, meta-analyses of published trials report very large psilocybin antidepressant effect sizes (Cohen's *d* > 0.8, 95% CI −1.285 to −0.367),^39^ but these are likely inflated as a result of the small sample sizes in each study. Still, we have a power of 0.80 to detect effect sizes of 0.96 and 0.91, when comparing changes on the MADRS over 2 days and 4 weeks, respectively, between the two psilocybin groups and the risperidone plus placebo group. The power is reduced to 0.67 for an effect size of 0.80 and 0.33 for an effect size of 0.5. The power and sample size calculations assume a 10% drop-out rate, two-tailed tests and significance of 0.05.

#### Statistical analysis plan

Characteristics of the trial sample will be summarised descriptively with means (s.d.), medians and ranges (minimum, maximum). Summary raw scores will also be presented at each assessment time both numerically and graphically.

All analyses will be conducted with a blinded grouping variable and under the intention-to-treat approach; the full information maximum likelihood method will be employed to tackle missing data. Diagnostic analyses and sensitivity analyses will be implemented to examine outliers, high influential cases, normality and missing-at-random assumptions. Standard frequency analysis will be completed for feasibility (recruitment, completion), safety and tolerability outcomes (adverse events).

For the analysis of clinical outcomes, the primary analytic strategy will be a generalised linear model to inspect group differences at the end-point. Individual-level random effects will be added to the model for longitudinal analysis (mixed-effects models). We will examine group differences in psychedelic effects as measured by the 5D-ASC, with treatment assignment as the primary predictor in the linear models. Differences in antidepressant effects will be examined by comparing changes in MADRS score across groups from baseline to 1 week after the dosing day (i.e. visit 5). Age, self-reported gender at birth and baseline MADRS total scores will be included as covariates. Mixed-effects model will be used to inspect change over time. For the exploratory aim, although not powered, a non-inferiority test will be conducted to examine if the psilocybin plus risperidone group demonstrates comparable effects on the MADRS to the psilocybin plus placebo group. Exact paired permutation *t*-tests will be used to determine whether PAP with psilocybin plus risperidone achieves a 50% reduction in MADRS score.

Dichotomous outcomes (e.g. response, remission, adverse effects) will be compared between groups, using a chi-squared test for assessments at a single time and generalised estimating equations for repeated assessments. Serious adverse events and drop-outs attributed to adverse events will be compared between groups, using a chi-squared test for assessments at a single time and generalised estimating equations for repeated assessments. We will also examine changes in secondary clinical measures by using the same general linear model framework. Point and confidence estimates will be the preferred measure to report group differences. Exploratory results will be reported uncorrected, as they are provided mainly for descriptive purposes and do not represent primary outcomes. The trial will be conducted and reported as per the CONSORT statement for RCTs.^40^

#### Subgroup analysis

Subgroup analysis will be completed to compare treatment effects between groups formed by sex (self-reported) and gender, by adding interactions with treatment to the regression models. Results for male and female gender will be reported separately irrespective of whether self-reported gender is a significant mediator. Exploratory analyses will compare responders and non-responders to identify potential clinical and demographic factors associated with clinical improvement.

Final statistical analysis will be completed only at the end of the trial. There will be no interim analysis.

### Safety monitoring

Adverse events monitoring and data collection will begin from the time informed consent is obtained until the final study visit (visit 8 on day 28). All laboratory tests, including blood work, urine samples and electrocardiograms, will be checked before randomisation. If any safety issues are identified that make a potential participant unsuitable for trial participation, they will not be randomised. The Columbia-Suicide Severity Rating Scale^41^ will be used to assess suicide potential or tendency as a study entry criteria, and will be monitored throughout the study at each visit to help rapidly identify this potential serious adverse event and intervene appropriately (e.g. further specialised assessment and hospital admission if needed). If a participant endorses suicidal ideation, the study team will use a published Suicide Risk Management Protocol to reduce suicide risk.^42^

The study team will be accountable to an independent Data Safety Monitoring Board (DSMB), which will comprise a chair with expertise in clinical trials, a biostatistician and one clinician with expertise in safety monitoring. The governance of the DSMB will be outlined in a charter agreed upon by all of its members. Guidelines provided by Health Canada will be followed for the monitoring and conduct of the study, to ensure the safety of participants and validity and integrity of study data. The DSMB will meet annually or more frequently when necessary. If needed, the DSMB will have access to unmasked data. The DSMB will make its recommendations to the Principal Investigator and Trial Steering Committee.

### Trial Steering Committee

A Trial Steering Committee will offer overall supervision and monitoring of the conduct of the trial. The committee will be independent of the study funders and the supplier of psilocybin. It will monitor the overall trial progress and conduct when advising on scientific issue. The committee will comprise a patient representative and three external experts in clinical trials, of whom at least one will have expertise on psilocybin. The committee will reflect and act, as suitable, upon the recommendations of the DSMB for deciding whether the trial needs to be stopped on grounds of safety. The committee will meet quarterly to ensure that the standards set out in the Guidelines for Good Clinical Practice are met, to monitor the progress of the study and adherence to the study protocol and to advise on ethical issues.

### Data protection and confidentiality

All research activities will be conducted in as private a setting as possible. All clinical trial-related documents and data will be held in strict confidence and stored at CAMH or on CAMH servers, and institutional policies and procedures will be followed to ensure participant privacy and confidentiality. Any study documentation with identifying information, such as signed informed consent forms, will be kept in a separate locked cabinet with access only to study personnel authorised by the Principal Investigator. Computer-based files/data will be entered into password-secured databases, and paper-based files will be stored in a secure and locked location. These data will only be accessible to study personnel and they will abide to confidentiality regulations of the REB. All computerised data will be coded, encrypted and password protected.

### Reporting

Outcomes will be reported according to CONSORT reporting guidelines.^43^

#### Ethical considerations

The authors assert that all procedures contributing to this work comply with the ethical standards of the relevant national and institutional committees on human experimentation and with the Helsinki Declaration of 1975, as revised in 2013.^44^ All procedures involving human participants were approved by the CAMH REB (approval number #080-2022, protocol version #2.0, approved on 22 February 2023).

The trial was registered with Clinicaltrials.gov (identifier: NCT05710237) on 2 February 2023 (https://clinicaltrials.gov/ct2/show/NCT05710237).

### Timeline

The anticipated start date for recruitment is May 2023 with a total project duration expected to last 36 months. Following a 6-month start-up period, including hiring, obtaining psilocybin and receiving a Health Canada Section 56 exemption to use psilocybin for scientific purposes, the study team will recruit approximately two to three participants per month over a period of 24 months. Study interventions and follow-up assessments will be completed by month 28. This leaves approximately 6 months for data analysis and reporting, which will be completed at month 36.

In total, there are a minimum of 12 study visits (eight study visits and a minimum of four check-in visits during the washout period). There may be more study visits scheduled at the discretion of the study team or the participant. The screening and washout period will take approximately 4–6 weeks, and the randomised part of the trial will last 4 weeks. All study visits will take place over the span of 8–10 weeks.

## Discussion

Depression affects nearly 280 million people globally,^45^ with up to a third of these cases being treatment resistant.^1^ It is estimated that approximately 30% of individuals suffering with TRD attempt suicide at least once in their lifetime.^46^ The high prevalence of TRD, and the disability and morbidity associated with it, make developing new effective treatments imperative. Several studies suggest that psilocybin in combination with therapy has therapeutic potential in the treatment of TRD.^9–13^ However, accessibility to PAP is limited by the time, specialised knowledge and financial resources required to offer it. Although the role of psychological support/therapy is out of the scope of the present trial, the results will inform future studies assessing this component of the intervention, particularly if the combination of psilocybin and risperidone demonstrates a clinical signal for antidepressant response in the absence of psychedelic effects. Also, given the potent psychedelic effects of psilocybin, some patients with depression may not be suitable candidates for this approach (e.g. those with a personal or family history of psychosis), and other patients may be reluctant taking psilocybin because of concerns about a negative psychedelic experience (a ‘bad trip’).

To our knowledge, this study is the first to investigate in humans whether psychedelic effects are required for the therapeutic efficacy of psilocybin in the treatment of TRD. Findings from this study will be both scientifically and clinically impactful. Its results will advance our understanding of the biological mechanisms underlying the antidepressant effects of psilocybin. Currently, they are presumed to be mediated through 5-HT2A receptor agonism, although this hypothesis is contrary to evidence that implicates downregulation of 5-HT receptors as a mediation pathway for successful treatment of MDD.^47,48^ If our study confirms that, as in animal models,^23^ psilocybin in combination with risperidone improves symptoms in humans with depression, this suggests that other mechanisms of action are responsible for this antidepressant effect. Possible alternative mechanisms of action for psilocybin include interactions with other 5-HT receptors,^19^ effects on synaptic connectivity,^49^ actions on brain-derived neurotrophic factor^50^ or modulation of inflammatory processes.^51^ Elucidating the mechanisms by which psilocybin exerts its antidepressant effects will inform novel therapeutic development in TRD. Furthermore, many of the practical barriers to the scale-up of PAP could be reduced or eliminated if psilocybin's psychedelic effects are not needed for its antidepressant efficacy. If this study demonstrates that administering psilocybin in combination with risperidone is feasible, safe and potentially effective at reducing symptoms of TRD, it will inform the design of a larger RCT to establish the efficacy of ‘non-psychedelic’ psilocybin in TRD.

## Data Availability

Data that support the findings of this study will be made available from the corresponding author, M.I.H., upon reasonable request after publication of findings.
